# Inhibition of FKBP51 induces stress resilience and alters hippocampal neurogenesis

**DOI:** 10.1038/s41380-022-01755-9

**Published:** 2022-09-14

**Authors:** Martin G. Codagnone, Nirit Kara, Anna Ratsika, Brunno Rocha Levone, Marcel van de Wouw, Laura A. Tan, Jacobi I. Cunningham, Connie Sanchez, John F. Cryan, Olivia F. O’Leary

**Affiliations:** 1 Department of Anatomy and Neuroscience, University College Cork, Cork, Ireland; 2 APC Microbiome Ireland, University College Cork, Cork, Ireland; 3 Alkermes, Inc., Waltham, MA USA; 4 Present Address: Instituto de Biología Celular y Neurociencia “de Robertis” IBCN (UBA-CONICET), Buenos Aires, Argentina

**Keywords:** Neuroscience, Depression, Drug discovery

## Abstract

Stress-related psychiatric disorders such as depression are among the leading causes of morbidity and mortality. Considering that many individuals fail to respond to currently available antidepressant drugs, there is a need for antidepressants with novel mechanisms. Polymorphisms in the gene encoding FK506-binding protein 51 (FKBP51), a co-chaperone of the glucocorticoid receptor, have been linked to susceptibility to stress-related psychiatric disorders. Whether this protein can be targeted for their treatment remains largely unexplored. The aim of this work was to investigate whether inhibition of FKBP51 with SAFit2, a novel selective inhibitor, promotes hippocampal neuron outgrowth and neurogenesis in vitro and stress resilience in vivo in a mouse model of chronic psychosocial stress. Primary hippocampal neuronal cultures or hippocampal neural progenitor cells (NPCs) were treated with SAFit2 and neuronal differentiation and cell proliferation were analyzed. Male C57BL/6 mice were administered SAFit2 while concurrently undergoing a chronic stress paradigm comprising of intermittent social defeat and overcrowding, and anxiety and depressive -related behaviors were evaluated. SAFit2 increased neurite outgrowth and number of branch points to a greater extent than brain derived neurotrophic factor (BDNF) in primary hippocampal neuronal cultures. SAFit2 increased hippocampal NPC neurogenesis and increased neurite complexity and length of these differentiated neurons. In vivo, chronic SAFit2 administration prevented stress-induced social avoidance, decreased anxiety in the novelty-induced hypophagia test, and prevented stress-induced anxiety in the open field but did not alter adult hippocampal neurogenesis in stressed animals. These data warrant further exploration of inhibition of FKBP51 as a strategy to treat stress-related disorders.

## Introduction

Up to 30% of patients with depression, a stress-related psychiatric disorder, remain nonresponsive or poorly responsive to available antidepressant treatments [1, 2]. Despite the pressing need for more effective treatments, only a few novel targets have emerged over the past decades [3].

One of the key players in the pathogenesis of stress-related disorders is thought to be severe or chronic activation or dysfunction of the hypothalamic-pituitary-adrenal (HPA) axis. Failure of antidepressant treatment to normalize the HPA axis response to dexamethasone suppression has been associated with antidepressant treatment resistance in depressed patients [4]. Human brain imaging studies have revealed that depression is associated with volumetric reductions in the hippocampus [5–8]. In parallel, studies in animals examining the impact of stress on the hippocampus have shown that either stress [9–13] or chronic exposure to the stress hormone corticosterone, induces hippocampal atrophy as characterized by a retraction of dendritic processes and the inhibition of hippocampal neurogenesis [14–18]. Furthermore, human postmortem analysis revealed that hippocampal neural progenitor cells (NPCs) are increased by chronic antidepressant treatment [19, 20]. Animal studies suggest that hippocampal neurogenesis is required for at least some of the effects of antidepressants on antidepressant-like behavior and normalization of HPA axis hyperactivity [21, 22]. Thus, regulation of adult hippocampal neurogenesis, perhaps through the glucocorticoid receptor (GR), which is also the key player in exerting negative feedback on HPA axis activity, is thought to be relevant to both the pathogenesis and treatment of stress-related psychiatric disorders [15, 18, 23, 24]. Therefore, modulators of GR-mediated feedback are potential targets for antidepressant drug development.

One such modulator of GR sensitivity is one of its co-chaperones, the immunophilin FK506-binding protein 51 (FKBP51) [25]. FKBP51 decreases GR affinity for its ligand, cortisol/corticosterone, and impedes its translocation to the nucleus [26]. In humans, genetic variations of FKBP51 have been identified as a risk factor for the development of post-traumatic stress disorder (PTSD), enhanced recurrence of depressive disorders, and faster antidepressant treatment response [27–33].

Recently, highly selective inhibitors of FKBP51 were discovered, opening a new window of opportunity for validation of pharmacological FKBP51 inhibition as a therapeutic target for stress-related psychiatric disorders [34]. Indeed, pharmacological inhibition of FKBP51 with SAFit2 improved rodent coping behavior in acute stressful challenges [34, 35]. Nonetheless, little is known about the potential use of SAFit2 in the context of promoting resilience in chronic stress models and whether it affects hippocampal neurogenesis. Thus, the aim of this study was to determine if inhibition of FKBP51 by SAFit2 promotes hippocampal neuron outgrowth, hippocampal neurogenesis and resilience to stress-induced behavioral changes.

## Materials and methods

Detailed methods can be found in supplemental material.

### Animals

All procedures were conducted with approval from the Animal Experimentation Ethics Committee (AEEC) at University College Cork and the Health Products Regulatory Authority, under project authorization number AE19130/P076, in accordance with the recommendations of the Directive 2010/63/EU.

### Drugs

SAFit2 ((2 S)-1-[(2 S)-2-cyclohexyl-2-(3,4,5-trimethoxyphenyl)acetyl]-2-piperidinecarboxylic acid, (1 R)-3-(3,4-dimethoxyphenyl)-1-[3-[2-(4-morpholinyl)ethoxy]phenyl]propyl ester), a highly selective FKBP51 inhibitor was synthetized by Alkermes as reported by Gaali et al. [34]. Further details on the specificity of this drug and ability to cross the blood brain barrier are provided in supplemental methods section. Fluoxetine hydrochloride (Sigma-Aldrich, UK) and all other chemicals were commercially available.

#### Experimental design of in vitro experiments

##### Primary hippocampal culture

Embryonic neuronal culture was prepared from E18 C57BL/6 mice (Biological Services Unit, UCC) according to Gaali et al. [34], and cells were treated for 48 h with SAFit2 (1, 10, 100, 250, 500, or 1000 nM) dissolved in dimethyl sulfoxide (DMSO, Sigma) to a final concentration of 1% (v/v) DMSO, brain derived neurotrophic factor (BDNF; Sigma, 40 ng/mL) in saline or 1% (v/v) DMSO, before fixation. Each condition was done in triplicate and the experiment was repeated three times.

#### Hippocampal NPC cultures

For neurosphere cultures, postnatal day 28 male Sprague Dawley rat (Biological Services Unit, UCC) hippocampal neural progenitor cells (NPCs) were isolated and cultured as previously reported [18]. At DIV 4, neurospheres were dissociated. To investigate the effects of SAFit2 on cell proliferation, cells were seeded in Poly-D-lysine coated (PDL) coverslips, exposed to 0.2 µM of bromodeoxyuridine (BrdU) in growth media and SAFit2 1, 10 or 100 nM or 0.1% DMSO (v/v) and allowed to proliferate for 4 h prior to fixation. To investigate the effects of SAFit2 on neuronal differentiation, NPCs were allowed to differentiate in PDL coated coverslips for 7 days in the presence of SAFit2 (1, 10 or 100 nM) or DMSO 0.1% (v/v) and then fixed as described below. Each condition was done in triplicate and the experiment was repeated three times.

### Immunocytochemistry

Cells were fixed with 4% paraformaldehyde (PFA; Fisher Scientific, Ireland), washed with PBS - 0.02% Triton X-100 (PBS-T) and blocked overnight with 5% donkey serum (DS; Sigma-Aldrich, UK). 18 h later, cells were incubated overnight in the appropriate primary antibody diluted in 5% DS 0.02% PBS-T: rat monoclonal anti-BrdU (1:100, ab6326, Abcam) or mouse monoclonal anti β3-tubulin (1:250, G7121, Promega). 18 h later, cells were washed and incubated for 2 h in their respective secondary antibody diluted in 5% DS 0.02% PBS-T: donkey anti-rat Alexa Fluor 594 (1:1000, A21209, Invitrogen) or donkey anti-mouse Alexa Fluor 488 (1:500, A-21202, Invitrogen). Cells were washed and then incubated in DAPI (1:1000, from a stock of 1ul/ml, Sigma-Aldrich, UK) diluted in 2% DS. Coverslips were mounted onto slides using DAKO Fluorescence mounting medium and slides were kept at 4 °C until imaging.

## Experimental design of in vivo experiments

### Effects of acute SAFit2 administration in the forced swim test and on plasma corticosterone

#### Drug administration and behavior

8-week old C57Bl6 male mice (Envigo, UK) were injected (i.p.) with either SAFit2 (20 mg/kg) or vehicle [4% (v/v) ethanol (Sigma-Aldrich, UK), 5% (v/v) PEG-400 (Fisher Scientific, Ireland) and 5% (v/v) Tween 80 (Sigma-Aldrich, UK) in saline] 16 or 1 h prior to test. The forced swim test (FST) was performed as previously described [36, 37]. The behavior was digitally recorded, and the time spent immobile during the last 4 min of the 6-min trial was used as treatment effect. Immobility was defined as the total absence of movement except slight motions to maintain the head above the water.

#### Plasma corticosterone

Prior to the start of the FST, and 30 and 90 min after the FST had started, 40 μl of whole blood was collected from the end of the tail. Plasma was removed and kept at –80 °C until analysis. Plasma corticosterone was measured using a commercial ELISA kit (ADI-901-097, Enzo) according to the manufacturer´s protocol. Light absorbance was read with a multi-mode plate reader (Synergy HT, BioTek Instruments, Inc.) at 405 nm. Samples were analyzed in duplicate.

### Effects of chronic SAFit2 or fluoxetine on stress-induced behavior

The experimental design is summarized in Fig. 5A.

#### Drug administration

Stressed and non-stressed C57Bl6 male mice were administered SAFit2 (20 mg/kg; i.p.) or vehicle (i.p.) twice a day or fluoxetine (10 mg/kg, i.p.; Sigma-Aldrich, UK) once a day. SAFit2 was dissolved in vehicle (see previous section) and fluoxetine in saline. There were five experimental groups: non-stressed animals injected with vehicle (*n* = 9); non-stressed animals injected with SAFit2 (*n* = 9); stressed animals treated with vehicle (*n* = 10) or stressed animals treated with SAFit2 (*n* = 10); and stressed animals treated with fluoxetine (*n* = 10).

#### Chronic stress

The stress paradigm including social defeat and overcrowding was carried out during 5 weeks as previously described [38, 39]. Non-stressed animals were handled daily. No stress took place on the days that animals underwent behavioral testing.

#### Behavioral tests

Behavioral assessment took place during the last two weeks of the experiment (Fig. 5A). Behavioral tests were performed in the following order: (1) social interaction [39]; (2) open field [39]; (3) novelty induced hypophagia [40], (4) female urine sniffing [38]; (5) light-dark box [41] and (6) FST [37]. Tests were carried out as previously described and detailed in Supplementary material.

#### Plasma corticosterone measurement

Blood was collected on days 17 and 35 of the experiment, as detailed in Supplementary material. On day 35 collection took place 5 min before and 30, 60 and 120 min after the FST. Preparation of plasma and the measurement of corticosterone was conducted as described above.

#### Transcardial perfusion and brain sectioning

On day 35, animals were deeply anaesthetized with sodium pentobarbital (90 mg/kg), transcardially perfused with PBS followed by 4% Paraformaldehyde (PFA) and brains were post-fixed in cold 4% PFA for 24 h at 4 °C. Fixed brains were then sectioned at 35 µm using a Leica CM1900 cryostat and stored in cryoprotectant at –20 °C until further processing.

#### Immunohistochemistry of hippocampal sections

Dorsal (–1.55 mm to –2.03 mm to Bregma) and ventral (–2.79 mm to –3.27 mm to Bregma) hippocampal sections were stained for DCX + neuronal cell bodies to assess immature neuron formation in this area. Sections were blocked with 10% DS in 0.3% PBS-T for 2 h at room temperature. Sections were then incubated in primary rabbit anti-DCX (1:500, ab18723, Abcam) in 2% DS, 0.3% PBS-T, overnight at room temperature. The next day, sections were washed in 0.3% PBS-T and incubated with donkey anti-rabbit secondary antibody (1:200, Alexa Fluor 488) in 2% DS, 0.1% PBS-T for 90 min at room temperature and protected from light. Sections were then washed in PBS and counterstained with DAPI for 5 min. Lastly, sections were washed in PBS, mounted on glass slides and cover-slipped with PVA DABCO mounting media. Slides were stored at 4 °C and covered from the dark until imaging.

### Fluorescence microscopy and image analysis

Slides were viewed under an Olympus BX53 upright microscope and photomicrographs of immunopositive cells were captured at ×20 or ×40 magnification using an Olympus DP71 camera and CellSens™ capture software. Immunocytochemistry: for each treatment condition, 3 coverslips were imaged and 5 fields of view were randomly captured per coverslip, and each experiment was repeated 3 times. Immunohistochemistry: for each treatment group, 3 hippocampal sections were randomly captured per brain area (dorsal or ventral hippocampus) and 5–6 animals were used per experimental group. The NeuronJ plugin of ImageJ 1.44 [42] was used for semiautomated tracing of individual neurons in calibrated images. Investigators who performed quantification were blinded to treatment groups. Quantification details are provided in supplemental material.

### Statistical analysis

Data is shown as mean + SEM. Statistical analysis was performed using IBM SPSS Statistics 26. In vitro data and the latency to social defeat were analyzed using one-way ANOVA, followed by Tukey post hoc test for group-wise comparisons when appropriate. Student’s *t* test was used to analyze the in vivo acute SAFit2 administration experiment. Kruskal-Wallis non-parametric test followed by Dunn’s comparison was used to analyze the behavioral data of the chronic stress and SAFit2 administration in the in vivo experiment. DCX + cell bodies per section of the dentate gyrus were analyzed using two-way ANOVA followed by Tukey post hoc test for group-wise comparisons when appropriate. For all comparisons, *p* < 0.05 was the criterion used for statistical significance.

## Results

### SAFit2 increased neurite length and complexity in vitro

**Fig. 1:**
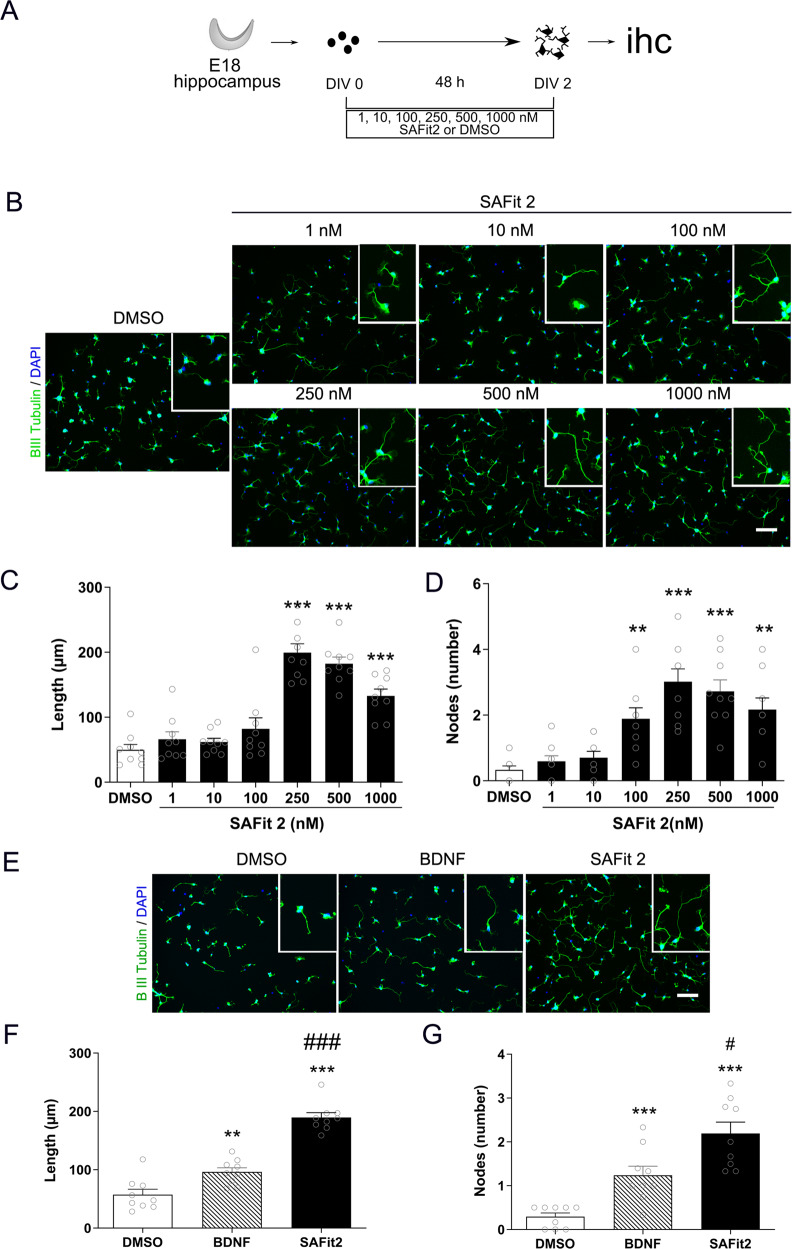
Effect of SAFit2 on hippocampal neurite outgrowth. Hippocampal neurons were isolated from E18 mice and exposed 48 h to SAFit2 or DMSO before fixation and beta II tubulin immunohistochemistry (**A**). SAFit2 increased neurite length, insets show ×2 magnifications (**B**). Quantification revealed a dose-dependent increase in neurite length (**C**) and nodes (**D**). Comparison to BDNF (40 ng/mL) confirmed neurotrophic effect of SAFit2 (500 nM) (**E**). Quantification revealed that SAFit2 induced a larger increase in neurite length and nodes (**F**, **G**). Results correspond to three independent cultures with triplicates in each (mean + SEM, *n* = 9 per group). DIV, day in vitro; ihc, immunohistochemistry; BDNF, brain-derived neurotrophic factor. *p < 0.05, **p < 0.01, ***p < 0.001 vs DMSO, #*p* < 0.05, ###*p* < 0.001 vs BDNF by one-way ANOVA with Tukey post hoc test.

We first evaluated the effect of 48 h SAFit2 exposure on neurite length and branching complexity in primary hippocampal neuronal cultures (Fig. 1A). ANOVA revealed a significant effect of SAFit2 on neurite length (Fig. 1C, F[6,55] = 38.556, *p* < 0.001). Subsequent post hoc analysis revealed a concentration-dependent effect whereby 250, 500, and 1000 nM of SAFit2 significantly increased neurite length (all *p* < 0.001). Lower concentrations (1 nM, 10 nM) had no effect (*p* = 0.911 and *p* = 0.980, respectively). Furthermore, ANOVA revealed that SAFit2 also increased number of branching nodes (F(6,55) = 14.443, *p* < 0.001; Fig. 1D). Specifically, 100, 250, 500 and 1000 nM of SAFit2 increased the number of branching nodes (100 nM *p* = 0.006, all others *p* < 0.001).

The neuritotrophic effect of SAFit2 was also compared to the positive control BDNF, (40 ng/mL), that is known to increase neuronal outgrowth in a similar experimental setting [43] (Fig. 1E). ANOVA revealed an effect of treatment on neurite length (F(2,24) = 68.492, *p* < 0.001; Fig. 1F) and number of branching nodes (F(2,24) = 22.982, *p* < 0.001; Fig. 1G). Tukey post hoc test revealed that 500 nM of SAFit2 increased neurite length and the number of branching nodes to a greater extent than BDNF (*p* < 0.001 vs DMSO for length and nodes; *p* < 0.001 and *p* = 0.006 vs BDNF for length and nodes, respectively). In sum, SAFit2 promoted neurite outgrowth to a degree comparable to a known neurotrophin.

### SAFit2 increased hippocampal neurogenesis in vitro and facilitated morphological development of newly born neurons

**Fig. 2:**
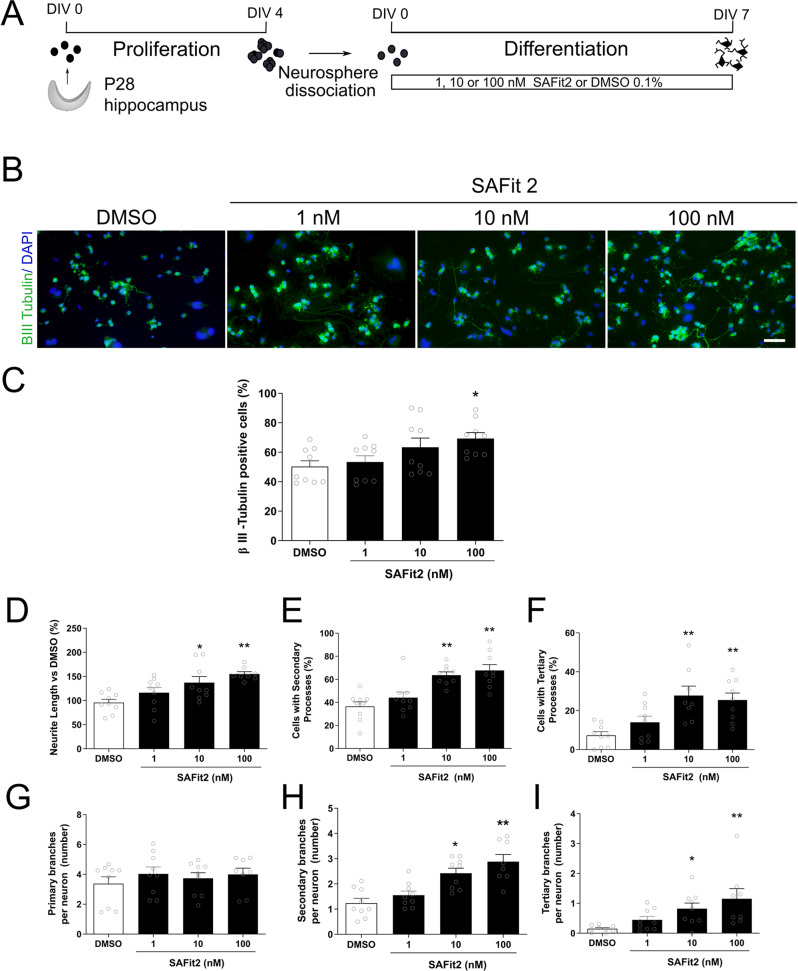
Effect of SAFit2 on hippocampal NPC differentiation. Hippocampal NPCs were isolated from P28 male rats, grown for four days as neurospheres, then dissociated and kept for 7 days under differentiation conditions while exposed to 1, 10, or 100 nM SAFit2 or DMSO (**A**). SAFit2 increased NPC differentiation into beta III tubulin positive cells and the neurite length (**B**). Quantification revealed that 100 nM significantly increased the amount of beta III tubulin positive cells (**C**). Quantification revealed that SAFit2 induced a dose-dependent increase in neurite length (**D**) and an increase in the proportion of neurons with secondary and tertiary processes (**E**, **F**). SAFit2 induced no change in the number of neurons with primary branches (**G**) but increased those with secondary and tertiary branches (**H**, **I**). Results correspond to three independent cultures with triplicates in each (mean + SEM, *n* = 9 per group). NPC neuroprecursor cells, DIV day in vitro, P postnatal day. *p < 0.05, **p < 0.01, ***p < 0.001 vs DMSO, by one-way ANOVA with Tukey post hoc test.

To investigate the effect of SAFit2 on neurogenesis, hippocampal NPCs were grown in vitro as neurospheres for four days before being dissociated and then grown under differentiation conditions for 7 days in the presence of various concentrations of SAFit2 (1, 10 and 100 nM; Fig. 2A). SAFit2 increased the number of cells that differentiated into neurons (beta III tubulin positive cells) (H(3) = 8.397, *p* = 0.038; Fig. 2B) suggesting increased neurogenesis. Dunn’s comparison revealed that only the highest concentration tested, 100 nM, increased neurogenesis [100 nM, p = 0.006; 10 nM *p* = 0.102; 1 nM *p* = 0.447; Fig. 2C].

SAFit2 significantly increased neurite outgrowth of newly born neurons (F(3,31) = 7.328, *p* = 0.001; Fig. 2B). Tukey post hoc comparison showed that 10 nM (*p* = 0.017) and 100 nM (*p* = 0.001) significantly increased neurite length (Fig. 2D). Furthermore, SAFit2 significantly increased morphological neuronal maturation as evidenced by the increased numbers of new neurons with secondary (F(3,32) = 12.479, *p* < 0.001) and tertiary branches (ANOVA, F (3,32) = 5.686, *p* = 0.003) (Fig. 2E, F). Specifically, Tukey post hoc comparison revealed that 10 nM and 100 nM increased the number of neurons with secondary (both *p* < 0.001) and tertiary branches (*p* = 0.011 and *p* = 0.007, respectively) (Fig. 2F, G, respectively).

While SAFit2 had no effect on the number of primary neurites per neuron (F(3,31) = 0.500, *p* = 0.685; Fig. 2G), it significantly increased the number of secondary and tertiary ramifications per neuron (F(3,31) = 12.843, *p* < 0.001 and H(3) = 16.373, *p* = 0.001 respectively; Fig. 2H, I). Specifically, Tukey post hoc comparison showed that both 10 nM and 100 nM increased the number of secondary (*p* = 0.002 and *p* < 0.001, respectively) and tertiary (*p* = 0.002 and *p* < 0.001, respectively) branches per neuron. Under these assay conditions the antidepressant, fluoxetine, did not affect morphological development of new hippocampal neurons (Supplementary Fig. 1B).

### SAFit2 increased hippocampal cell proliferation in vitro

**Fig. 3:**
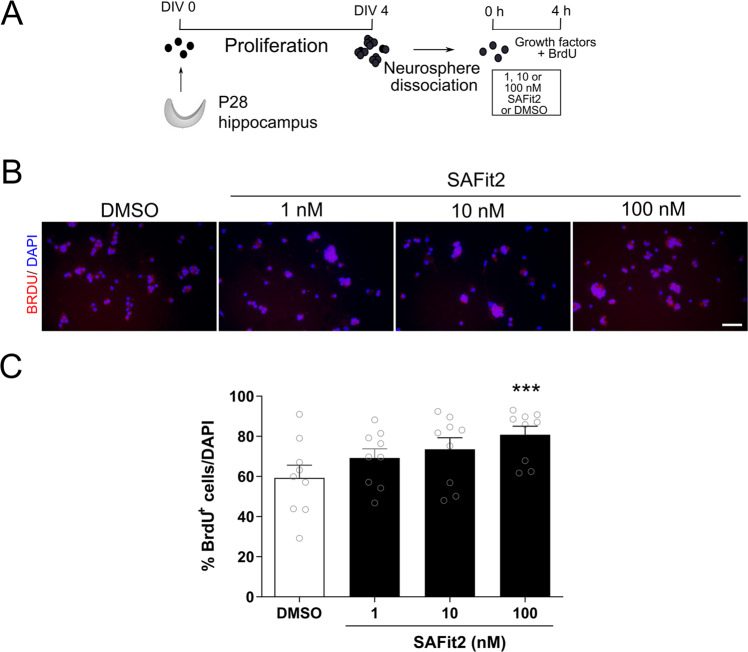
Effect of SAFit2 on hippocampal NPC proliferation. Hippocampal NPC were isolated from P28 male rats, grown for four days as neurospheres, then dissociated and kept in a proliferative environment with 0.02% BrdU for 4 hs while exposed to 1, 10 or 100 nM SAFit2 or DMSO (**A**). Representative images of BrdU staining (**B**). Quantification of nuclear BrdU-positive NPCs revealed that 100 nM SAFit2 significantly increased NPC proliferation (**C**). Results correspond to three independent cultures with triplicates in each (mean + SEM, *n* = 9 per group). NPC neuroprecursor cells, DIV day in vitro, P postnatal day. *p < 0.05 vs DMSO, by one-way ANOVA with Tukey post hoc test.

We investigated whether SAFit2-induced neurogenesis might be explained, in part, by increased NPC proliferation. Cells isolated from hippocampal neurospheres were exposed to BrdU and different concentrations of SAFit2 over a period of 4 h (Fig. 3A). ANOVA and subsequent Tukey post hoc comparison revealed that 100 nM SAFit2, the same concentration that increased neurogenesis, also increased proliferation as indicated by the increased number of BrdU positive cells (F(3,32) = 2.861, *p* = 0.052; post hoc, *p* = 0.035; Fig. 3C). Of note, fluoxetine did not increase hippocampal cell proliferation under these assay conditions (Supplementary Fig. 1A).

### Acute SAFit2 administration decreased immobility in the FST in a time-dependent manner

**Fig. 4:**
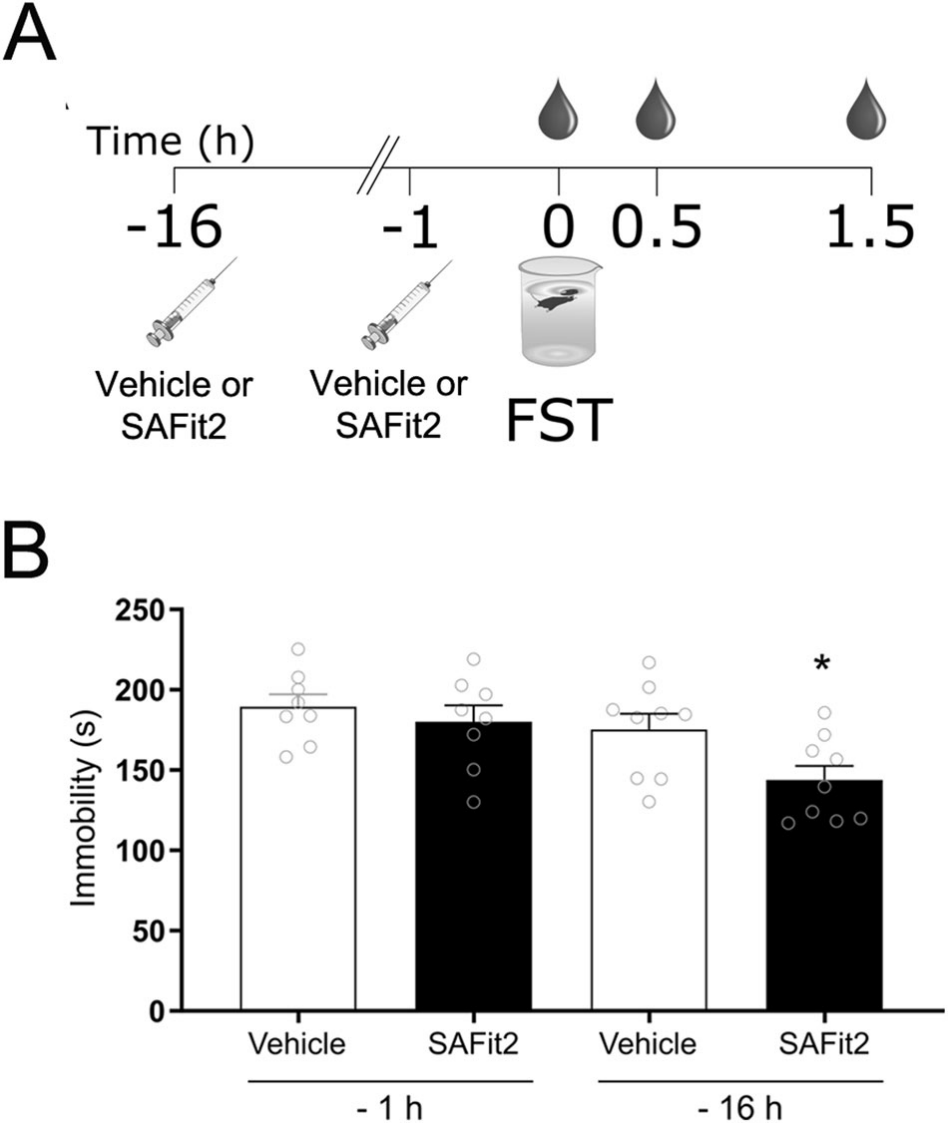
Time dependent action of SAFit2 in the forced swim test (FST). SAFit2 (20 mg/kg) or vehicle was administered i.p. 16 or 1 h prior to FST. Blood was collected before the behavioral testing and 30 and 90 min after (**A**). SAFit2 decreased the time spent immobile in FST when administered 16 h before the test but not 1 h (**B**). mean + SEM, *n* = 7–8 per group, *p < 0.05 vs Vehicle by Student *t* test.

SAFit2 (20 mg/kg; i.p.) was administered at either 16 h or 1 h prior to the FST (Fig. 4A). SAFit2 decreased immobility when administered 16 h (t(16) = 2.425, *p* = 0.028) but not 1 h (t(15) = –0.044, *p* = 0.965; Fig. 4B) prior to the FST. Thus, this dose of SAFit2 was suitable to be investigated in a chronic dosing regimen and a chronic stress paradigm.

### Chronic administration of SAFit2 prevented stress-induced social avoidance

**Fig. 5:**
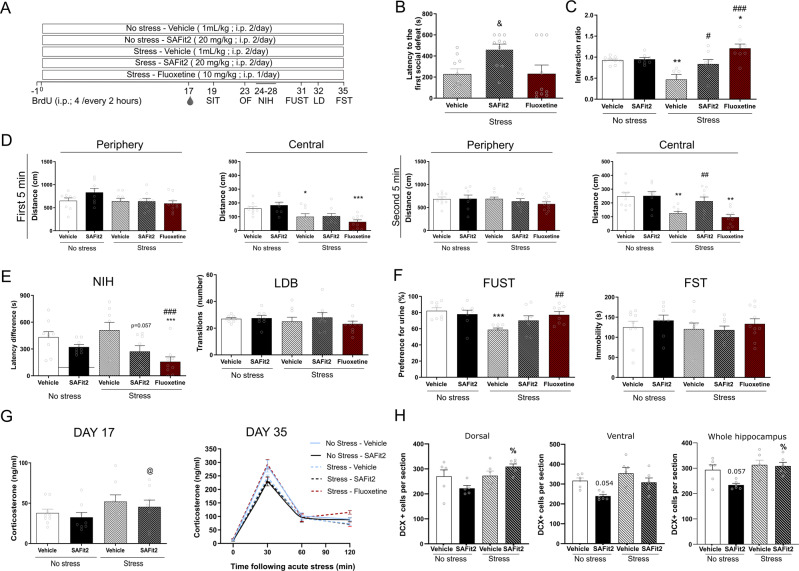
Behavioral and biochemical effect of chronic SAFit2 administration on chronic psychosocial stress. Schematic representation of experimental design (**A**). Briefly, SAFit2 (20 mg/kg) or vehicle was administered i.p. twice-a-day for five weeks while animals were subjected to social defeat and overcrowding. A separate group was administered fluoxetine i.p. (10 mg/kg once a day) while exposed to the stress. Blood was collected at days 17 and 35 of experiment and behavioral testing took place during the last two weeks of experiment. Administration of SAFit2 increased the latency to the first social defeat (**B**). Both SAFit2 and fluoxetine prevented the decrease in social interaction caused by chronic psychosocial stress (**C**). Stress induced a decrease in the distance traveled in the central area of the open field that was prevented by the administration of SAFit2 during the second 5 min (**D**). SAFit2 and fluoxetine decreased the latency difference in the NIH but neither stress nor drug treatment affected transitions in the LDB (**E**). Stress-induced decrease in preference for female urine was only prevented by the administration of Fluoxetine (**F**). Neither stress nor drug treatment affected immobility time in FST (**F**). At experimental day 17, there was no effect of chronic SAFit2 administration on chronic stress-induced increase in plasma corticosterone levels (**G**). At experimental day 35, there was no effect of chronic stress or chronic drug administration on acute stress-induced corticosterone levels (**H**). Quantification of immature DCX + new neurons in dorsal, ventral and whole hippocampus show a discrete effect of SAFit2. SIT, social interaction test; OF open field, LDB light-dark box, NIH novelty induced hypophagia. FUST female urine sniffing test, FST forced swim test. mean + SEM, *n* = 6–10 per group. % *p* < 0.05 by two-way ANOVA followed by Tukey post hoc test, & *p* < 0.05 by one-way ANOVA followed by Tukey post hoc test. *p < 0.05, **p < 0.01 ***p < 0.01 vs no stress–vehicle, and #*p* < 0.05, ###*p* < 0.001 vs stress-vehicle and @<0.01 vs no stress-SAFit2 by Kruskal-Wallis followed by Dunn’s comparison.

First, we investigated the effect of chronic SAFit2 in stress-induced social aversion, a phenotype induced by a chronic social defeat/overcrowding stress paradigm. ANOVA revealed a significant effect of treatment on the latency to the first social defeat (F(2,27) = 3.60, *p* = 0.041) (Fig. 5B). Post hoc Tukey test revealed that upon the first exposure to the aggressor, SAFit2 (*p* = 0.040) but not fluoxetine (*p* = 0.784), prolonged the latency to subordination. Kruskal-Wallis analysis revealed a difference in the social interaction ratio (H(4) = 19.020, *p* < 0.0001; Fig. 5C). Post hoc Dunn’s comparison revealed that both SAFit2 (*p* = 0.04) and fluoxetine (*p* < 0.001) prevented stress-induced social avoidance (stress-vehicle vs no stress-vehicle, *p* = 0.008). Moreover, stressed animals treated with SAFit2 showed similar social interaction levels to non-stressed animals (*p* = 0.554). Changes in social interaction were not due to stress- or treatment-induced changes in locomotor activity (data not shown).

### Chronic SAFit2 decreased stress-induced behavior in the novelty induced hypophagia test and prevented stress-induced behavior in the open field

Next, we investigated the effect of chronic SAFit2 in anxiety-related behaviors. The Kruskal-Wallis test revealed an effect of stress and treatment on distance traveled in the center of the open field (Fig. 5D). During the first 5 min of the test (H (4) = 18.270, *p* = 0.001), when the apparatus is novel to the animal, Dunn’s post hoc comparison revealed that stress decreased the distance traveled in the center of the open field (*p* = 0.037, stress-vehicle vs no stress-vehicle) and this was not prevented by either SAFit2 (*p* = 0.020 vs no stress–SAFit2) nor fluoxetine (*p* = 0.001 vs no stress-vehicle). However, during the second 5 min of the test (H(4) = 20.708, *p* < 0.001; Fig. 5D), when mice have habituated to the initial novelty of the apparatus, the stress-induced decrease in center activity (*p* = 0.006) was prevented by SAFit2 (*p* = 0.048 vs stress–vehicle and *p* = 0.431 vs no stress–SAFit2) but not fluoxetine (*p* = 0.507 vs stress-vehicle and *p* = 0.001 vs no stress-vehicle). There was no significant effect on activity in the periphery of the open field test in the two time intervals Kruskal-Wallis H(4) = 5.293, *p* = 0.258 and (H(4) = 3.845, *p* = 0.427) for first and second 5 min, respectively.

In the novelty-induced hypophagia test (Fig. 5E), Kruskal-Wallis revealed a significant effect of treatment on the latency to drink a sweet solution in a novel environment (H(4) = 16.081, *p* = 0.003). Although Dunn’s post hoc test did not show a statistically significant stress effect (*p* = 0.535, stress-vehicle vs no stress-vehicle), SAFit2 showed a strong trend to reduce latency to drink (*p* = 0.057 vs stress-vehicle) and fluoxetine (*p* < 0.001 vs stress-vehicle) reduced the latency to drink in a bright novel environment suggesting effects on stressed-induced behavior. There was no overall effect of stress and/or drug treatment in the number of transitions in the light-dark box (H(4) = 4.847, *p* = 0.303; Fig. 5E).

### Fluoxetine but not SAFit2 prevented stress-induced anhedonia in the FUST

Kruskal-Wallis revealed an effect of treatment and stress in female urine preference (H(4) = 13.871, *p* = 0.008; Fig. 5F). Subsequent Dunn’s post hoc comparison showed that stress significantly decreased preference to sniff female urine (*p* = 0.001 stress-vehicle vs no stress-vehicle). Fluoxetine prevented this stress-induced decrease (*p* = 0.01 vs stress-vehicle and *p* = 0.404 vs no stress-vehicle) while SAFit2 failed to show a statistically significance effect (*p* = 0.083 vs stress-vehicle and *p* = 0.315 vs no stress-SAFit2). Behavior in the FST was assessed on the last experimental day (Fig. 5F). Kruskal-Wallis revealed no effect of stress or treatment in this test (H(4) = 2.318, *p* = 0.678).

### SAFit2 does not alter stress-induced increases in plasma corticosterone concentrations

To gain further insight into the mechanism of SAFit2-induced stress resilience, we investigated plasma corticosterone levels. On experimental day 17 there was a significant chronic stress effect (H(3) = 12.7820, *p* = 0.005; Fig. 5G). Dunn’s post hoc analysis revealed an increase in corticosterone concentrations in stressed-SAFit2 treated animals compared to non-stressed SAFit2-treated animals (*p* = 0.002) but no significant increase in corticosterone in stressed vehicle-treated animals (*p* = 0.088). Chronic SAFit2 administration had no effect on corticosterone in stressed animals (*p* = 0.363 stress-vehicle vs stress-SAFit2).

On the last day of the experiment, we measured plasma corticosterone in response to the acute stress of the FST (Fig. 5G). There was a significant effect of prior chronic stress exposure and treatment on the plasma corticosterone response to the acute stress of the FST at 0, 30, and 120 min (H(4) = 10.969, *p* = 0.027; H(4) = 10.975, *p* = 0.027; H(4) = 16.712, *p* = 0.0027) with no effect at 60 min (H(4) = 0.174, *p* = 0.996). There was no effect of SAFit2 across timepoints but post hoc comparison revealed that fluoxetine-treated stressed groups induced a greater corticosterone response compared to vehicle treated-stressed group at 30 min and 120 min (*p* = 0.021 and *p* < 0.001).

Since we observed a significant effect of acute SAFit2 treatment on immobility in the FST, we also investigated whether this acute immobility response was associated with acute SAFit2 modulation of plasma corticosterone. Similar to chronic SAFit2 administration, there was no effect of acute SAFit2 on baseline or peak corticosterone levels (Supplementary Fig. 2). Taken together, under these experimental conditions, SAFit2 did not affect the reactivity of the HPA axis to stress.

### In vivo SAFit2 administration did not increase hippocampal DCX + cells

To evaluate effects of SAFit2 on neurogenesis in vivo, we investigated the number of immature (DCX+) new neurons in the hippocampus using immunohistochemistry (Supplementary Fig. 3). Two-way ANOVA revealed a significant main effect of stress in the number of immature neurons in the whole hippocampus but not by treatment or the stress x treatment interaction [Stress: F(1,24) = 9.262, *p* = 0.006; Treatment: F(1,24) = 4.320, *p* = 0.051; Stress x Treatment: F(1,24) = 3.182; *p* = 0.090; Fig. 5H]. Tukey post hoc comparison revealed that SAFit2 induced a tendency to decrease DCX + cells in non-stressed animals (*p* = 0.057) and stress increased the number of DCX + cells in SAFit2 treated animals (*p* = 0.013 vs non-stressed- SAFit2). Upon segregation along the longitudinal axis of the hippocampus, the dorsal hippocampus showed a significant main effect of stress and of the interaction [dHi: Stress: F(1,23) = 5.854, *p* = 0.026; Treatment: F(1,23) = 0.110, *p* = 0.744; Treatment x Stress: F(1,23) = 5.326, *p* = 0.032; Fig. 5H]. Post hoc comparison revealed that treatment with SAFit2 increased the number of DCX + cells in stressed animals (*p* = 0.019 non-stress SAFit2 vs stress- SAFit2). Similar to whole hippocampus, there was no significant effect of stress x treatment interaction in the ventral hippocampus (vHi), but an effect of stress and treatment [vHi: Stress: F(1,24) = 7.160, *p* = 0.015; Treatment: F(1,24) = 9.613, *p* = 0.006; Treatment x Stress: F(1,24) = 0.655, *p* = 0.428; Fig. 5H]. Post hoc comparison revealed a trend for SAFit2 to decrease DCX + cells in non stressed animals (p = 0.054 vs no stress- vehicle). Overall, these results show SAFit2- and stress- mediated complex regulation of adult hippocampal neurogenesis.

## Discussion

Here, we demonstrated that chronic administration of the selective FKBP51 inhibitor, SAFit2, prevented stress-induced social withdrawal and stress-related behaviors in an animal model of chronic stress. Moreover, we show for the first time that in vitro SAFit2 promotes proliferation and neuronal differentiation of hippocampal NPCs, as well as neurite outgrowth

One of the key features of the chronic stress model is the development of social avoidance [44]. Remarkably, SAFit2 prevented stress-induced social withdrawal and increased latency to adopt a defeat posture while it had no effect on stress-induced anhedonia in the FUST. Our data support the hypothesis that FKBP51 could mediate stress resilience [45–49] and are the first to provide evidence for a distinctive pro‐resilience action of SAFit2 in chronic stress.

Another behavioral domain relevant in stress-related psychiatric disorders is anxiety. Here we show SAFit2 prevented stress-induced behavior on NIH and shortened habituation to the aversive environment of the center of the open field. Though acute SAFit2 has been previously shown to have anxiolytic effects in the elevated plus maze [35], this is to our knowledge the first time anxiolytic effects of chronic SAFit2 administration in a chronic stress model have been shown. This effect of our chronic i.p. dosing study is consistent with anxiolytic effect of SAFit2 after acute systemic and central administration [35]. This suggests that no tolerance develops to the pharmacological effect. Overall, our data supports the importance of FKBP51 inhibition for anxiety-like behaviors and also challenges new evidence on the role of FKBP51 in anxiety regulation [50].

Contrary to what was shown by us and others in acute settings [35], but similar to acute treatment in Pöhlmann et al. [51] and chronic treatment in Balsevich et al*.* [52], chronic SAFit2 had no effect in the light-dark box test or the forced swim test. In parallel however, no effect of chronic stress was observed in these tests or on basal concentrations of corticosterone taken at the end of the experiment just prior to the FST which might have been a contributing factor to our data. This contrasts with corticosterone measurements taken at an earlier time point in the experiment which demonstrated that the chronic stress paradigm increased basal corticosterone after 17 days of chronic stress. This might be explained by the fact that exposure to the severity of the stress paradigm decreased over time as stressors were not applied on behavioral test days. In this context, the FUST, LDB and the FST were the last behavioral tests to be conducted with no stress effects observed in the LDB or FST and a modest stress effect observed in the FUST. Similarly, we found that chronic treatment with SAFit2 but not fluoxetine prevented stress-induced weight gain (which is a phenotype of this chronic stressor) but this effect tapered off when frequency of stressor exposure decreased (Supplementary Fig. 4). Interestingly, similar stress-induced changes in body weight have been shown in FKBP51 knockout animals [47] and mediobasal hypothalamus FKBP51 deletion was shown to be sufficient to increase body weight [53]. We did not observe any effects of chronic SAFit2 administration on body weight of non-stressed mice which is in contrast to what was reported in a 30-day SAFit2 administration experiment done by Balsevich et al. [52]. Since chronic stress and depression are highly associated with symptoms of metabolic syndrome [54], our findings warrant further investigation into FKBP51 as a molecular link between stress and metabolism.

The precise mechanisms underlying SAFit2-induced stress resilience remain to be elucidated. Although age- and region- specific FKBP51 mediated regulation of the HPA axis has been proposed [55] and decreased basal corticosterone levels and decreased HPA axis reactivity has been reported in male and female FKBP51 knockout mice [46, 56], the lack of effect of acute and chronic SAFit2 administration on basal and stress-induced corticosterone concentrations in our study suggest that modulation of the HPA axis is not mediating the behavioral effects of SAFit2. In addition, the temporality of the behavioral effect of acute SAFit2 in the FST (i.e. effective after 16 h but not 1 h post-administration) suggest that other more gradual processes are involved in the mechanism of SAFit2 action. GR/FKBP51 continue to be considered targets of antidepressants [57] but other mechanisms could underlie SAFit2 pro-resilience action [58].

Our in vitro data showed for the first time that SAFit2 promoted the proliferation and neuronal differentiation of rat postnatal hippocampal NPCs, suggesting that pharmacological inhibition of FKBP51 can increase hippocampal neurogenesis, a process hypothesized to be important in antidepressant action [20, 21, 59]. In addition, we found that SAFit2 increased neurite growth complexity of these new hippocampal neurons from postnatal NPCs. We also found that SAFit2 effectively promoted neurite outgrowth and complexity in embryonic primary hippocampal cell cultures and similar findings have been reported by others [34, 60]. However, these findings did not translate in vivo. The difference between in vivo and in vitro findings may be related to the species used (mice for in vivo and rat for in vitro), SAFit2 levels in vitro vs in vivo hippocampal levels, length of exposure to SAFit2 (7days in vitro vs 35 days in vivo) or differences in neurogenic niche in vitro vs in vivo which may influence NPC GR activity. Interestingly, in vivo there was a trend for SAFit2 to decrease DCX + cells under non-stress conditions however under stress conditions this effect was not apparent, suggesting an interaction between stress conditions and SAFit2 treatment in regulation of adult hippocampal neurogenesis.

Excluding direct action on HPA axis or hippocampal neurogenesis, the mechanism underlying stress-resilience effects of SAFit2 is likely complex as FKBP51 and its co-chaperone HSP90 are involved in numerous signaling pathways. SAFit2-mediated FKBP51 inhibition is expected to promote HSP90 and FKBP52 function [61]. It could be that stress resilience effects independent of GR-activity are mediated by HSP90. Normal functioning of this chaperone is needed for adequate neuronal polarization, FKBP51/HSP90 binding disruption by SAFit2 could lead to modulation of HSP90 activity with concomitant reduction of Akt and GSK3 activity [62, 63]. GSK3 inhibitors have antidepressant effects and reduction of GSK3 signaling pathway promotes resilience to social defeat stress [64]. AKT has also been implicated in the antidepressant activity of ketamine by activation of downstream mTOR signaling, a pathway that could be involved in SAFit2 behavioral effects [65]. Furthermore, another important pathway downstream of HSP90 that could be modulated by SAFit2 is GSK3 mediated autophagy, which has been linked to antidepressant activity [66]. Previous findings suggest that SAFit2-induced neuronal differentiation could be mediated by FKBP52 upregulation, resulting in the activation of a neuronal ERK cascade pathway that drives cytoskeleton rearrangement [60, 67]. Inhibition of ERK signaling pathway in hippocampus and prefrontal cortex cause depression-like behavior [68]. Thus, SAFit2- mediated activation of this pathway might explain SAFit2 antidepressant-like behavior and in vitro neuronal outgrowth. Another consideration is the NFkB signaling pathway, a critical mediator of the behavioral effects of chronic stress [69]. Higher FKBP52/FKBP51 ratio can promote NF-kB signaling and gene transcription that has been linked to modulation of depressive-like behavior [70]. More research is needed to elucidate the mechanisms underlying SAFit2 pro-resilience action.

In summary, we have shown that SAFit2 administration promotes a distinctive pro-resilience phenotype in an animal model of chronic stress, preventing social avoidance and anxiety-like behavior. In vitro, SAFit2 promoted neuronal outgrowth and hippocampal neurogenesis but this was not observed in vivo suggesting that alternate mechanisms are involved in stress resilience effects of SAFit2. We suggest that FKBP51 inhibition warrants further research on its therapeutic potential for the treatment of stress-related psychiatric disorders.

## Supplementary information

Supplemental material
