# Supplementary material for: Inhibition of FKBP51 induces stress resilience and alters hippocampal neurogenesis
Source: Mol Psychiatry. 2022 Sep 14;27(12):4928–38. doi: 10.1038/s41380-022-01755-9 (PMC9763121; doi:10.1038/s41380-022-01755-9)
Supplement: Supplementary file 1 — Supplemental material [file 41380_2022_1755_MOESM1_ESM.docx]

## Supplemental material

## Detailed Materials and Methods

*Animals*

All procedures were conducted with approval from the Animal Experimentation Ethics Committee (AEEC) at University College Cork and the Health Products Regulatory Authority, under project authorization number AE19130/P076, in accordance with the recommendations of the Directive 2010/63/EU. For *in vitro* experiments, female C57BL/6 mice at embryonic day (E) 18 and postnatal day (P) 28 Sprague Dawley male rats were purchased from Biological Services Unit (Ireland). For *in vivo* experiments, male C57BL/6 and CD-1 mice (Envigo, UK) were housed in separate rooms at arrival. Room temperature was controlled (21 ± 2°C) and mice allowed to acclimatize for 7-days prior to initiation of experiments. Laboratory chow and water were provided ad libitum on a 12 / 12 h light/dark cycle (lights on at 7:30 am).

*Drugs*

To specifically inhibit FKBP51 without collateral FKBP52 inhibition, SAFit2 ((2S)-1-[(2S)-2-cyclohexyl-2-(3,4,5-trimethoxyphenyl)acetyl]-2-piperidinecarboxylic acid, (1R)-3-(3,4-dimethoxyphenyl)-1-[3-[2-(4-morpholinyl)ethoxy]phenyl]propyl ester) , a highly selective induced fit FKBP51 inhibitor was synthetized as reported by Gaali *et al* (1). Detailed characterization of this inhibitor was made available by the authors, including molecular design and pharmacokinetic properties. SAFit2 binds to FKBP51 with high selectivity. It is more permeable to the blood-brain barrier than other inhibitors, brain/plasma ratio of 16.7% after 10 mg/kg i.p. injection (1). Furthermore, Hartmann et al (2) found that the anxiolytic effects of peripheral administration of SAFit2 could be mimicked by injecting the drug directly into the amygdala brain region. For its functional characterization, Gaali et al. (1) chose an *in vitro* approach, testing the effect of SAFit2 on N2a neurite outgrowth. Neurite outgrowth in N2a cell line had been reported to be a distinctive feature of FKBP51 knock-out (1, 3) showed that by using SAFit 2, a similar effect could be achieved. We validated this SAFit2 functional readout in Figure 1 in primary hippocampal cultures and brought additional novelty by comparing the effect of the drug to that of a known neurotrophic factor (BDNF).

Fluoxetine hydrochloride (Sigma-Aldrich, UK) and all other drugs were commercially available.

**Experimental design of *in vitro* experiments**

**Primary hippocampal culture**

At E18, pregnant C57BL/6 mice were euthanized by decapitation and embryos quickly removed by caesarean section. The hippocampi were dissected in Hank's balanced salt solution (HBSS) and cells prepared according to previous reports (1). Briefly, hippocampi were trypsinized (trypsin-EDTA, 0.25 %), mechanically dissociated and plated on poly-D-lysine-coated (Sigma; PDL, 1 mg/mL per 30 min) 13 mm-diameter glass coverslips (Corning Life Sciences, UK), at 5 × 10^4^ cells per well. The cell suspension was seeded in Neurobasal medium (Invitrogen, UK) containing 2 % (v/v) B27 (Invitrogen, UK), 0.5 mM GlutaMAX (Invitrogen, UK) and 0.1 % (v/v) penicillin/streptomycin (Invitrogen) and incubated in a humidified atmosphere at 37 °C and 5 % CO_2_. 4 h later, cells were randomly assigned to each experimental group and treated for 48 h with different concentrations of SAFit2 (1, 10, 100, 250, 500, 1000 nM) dissolved in dimethyl sulfoxide (DMSO, Sigma) to a final concentration of 1 % (v/v) DMSO, brain derived neurotrophic factor dissolved in saline (BDNF; Sigma, 40 ng/mL) or 1 % (v/v) DMSO, before fixation. Each condition was done in triplicate and the experiment was repeated three times.

**Preparation of neurosphere cultures**

Hippocampal neural progenitor cells (NPCs) were isolated and cultured as previously reported (2). Three postnatal day (P) 28 male Sprague Dawley (Biological Services Unit, UCC) per culture were decapitated and whole hippocampi collected in HABG solution [comprised of hibernate A (Invitrogen, UK) with 2 % (v/v) B-27 minus vitamin A (Invitrogen, UK) and 0.5 mM glutamax]. After enzymatic tissue dissociation (1 U/mL dispase, 2.5 U/mL papain and 250 mL DNase I) and centrifugation, the pellet was washed with HABG resuspended in 20 % percoll (Sigma-Aldrich, UK) in neurobasal-A (Invitrogen, UK). After 10 min centrifugation at 800 g, a wash with HABG solution, the pellet was resuspended in growth medium: neurobasal-A, 2 % (v/v) B-27 minus vitamin A , 0.5 mM Glutamax, 50 U/mL penicillin/streptomycin (Sigma-Aldrich, UK), 2 µg/ml heparin (Sigma-Aldrich, UK), basic fibroblast growth factor (bFGF, 20 ng/ml, Millipore, UK) and epidermal growth factor (EGF, 20 ng/ml, Sigma-Aldrich, UK). Viable cells were counted with haemocytometer/trypan blue (Sigma-Aldrich, UK) and seeded at a density of 10 x 10^4^ cells per well with 500 µl of medium in ultralow adherence 24-wells plates (Corning Life Sciences, UK), and allowed to proliferate as neurospheres for 4 days in vitro (DIV). At DIV 2, half of the media was replaced with fresh media.

At DIV 4, all neurospheres were collected and dissociated with 0.08 % trypsin (Sigma) in neurobasal-A. After 5 min dissociation was terminated by adding trypsin inhibitor (Sigma, 0.125 mg/ml) and DNase I (250 U/ml). The cell suspension was centrifuged at 300 g for 5 min. The pellet was resuspended in growth medium followed by mechanical dissociation. Viable cells were counted with hemocytometer/Trypan blue (Sigma Aldrich, UK) and seeded at a density of 5 x 10^4^ in a volume of 40 µl per well in a poly-D-lysine-coated coverslip (13 mm). After allowing 1 h for attachment of the NPCs to the previously poly-D-lysin coated coverslips, 460 µl proliferation or differentiation treatment media was added. Cells were randomly assigned to each experimental condition.

To investigate the effects of SAFit2 on cell proliferation, cells were exposed to 0.2 µM of bromodeoxyuridine (BrdU) in growth media and different concentrations of SAFit2 [1, 10 or 100 nM in a final concentration of 0.1 % (v/v) DMSO or DMSO 0.1 % (v/v)]. NPCs were allowed to proliferate for 4 h *in vitro* prior to fixation. To investigate the effects of SAFit2 on neuronal differentiation, NPCs were treated for 7 days with treatment media (neurobasal-A containing 2 % (v/v) B27, 0.5 mM glutaMAX and 0.1 % (v/v) penicillin/streptomycin) containing different concentrations of SAFit2 [1, 10 or 100 nM in a final concentration of 0.1 % (v/v) DMSO or DMSO 0.1 % (v/v)]. Half of the treatment media containing the relevant concentration of SAFit2 was replenished every other day. At DIV 7 cells were fixed as described below. Experiments investigating the effects of SAFit2 on cell proliferation and neuronal differentiation/neurogenesis were repeated three times and each experimental condition was done in triplicate within each experiment.

To investigate the effect of fluoxetine on NPC differentiation and proliferation, dorsal (dHi), intermediate (iHi) and ventral (vHi) hippocampus were dissected (4). To obtain a sufficient number of NPCs per independent experiment, each sub-region of 6 animals were pooled. NPCs were seeded at a density of 10 x 10^4^ cells per well and allowed to proliferate as neurospheres for 4 DIV similar to what was done for the SAFit2 experiment. To measure the effects of fluoxetine treatment on proliferation, NPCs were dissociated and allowed to proliferate in growth medium containing 0.2 µM BrdU and different concentrations of fluoxetine (FLX; 0, 0.1 or 1 µM) for 4 h in vitro prior to fixation. To measure the effects of fluoxetine treatment alone on neuronal differentiation and maturation, NPCs were dissociated and allowed to differentiate for 4 DIV in growth medium with different concentrations of fluoxetine (0, 0.1 or 1 µM).

*Immunocytochemistry*

Cells were washed with warm Hanks’ balanced salt solution (HBSS), fixed with 4 % paraformaldehyde (PFA; Fisher Scientific, Ireland) in 0.1 M PBS at room temperature for 20 min. Cells were then washed with PBS-0.02 % Triton X (PBS-T) and blocked overnight at 4ºC with 5 % donkey serum (DS; Sigma-Aldrich, UK) in PBS-T. 18 h later, cells were washed with 0.02 % PBS-T and incubated overnight at 4ºC in the appropriate primary antibody diluted in 5 % DS 0.02% PBS-T: rat monoclonal anti-BrdU (1:100, ab6326, Abcam) or mouse monoclonal anti β3-tubulin (1:250 G7121, Promega) or goat polyclonal anti-DCX (1:200, sc-8066, Santa Cruz)]. 18 h later, cells were washed and incubated for 2 h at room temperature in the respective secondary antibody diluted in 5 % DS 0.02% PBS-T: donkey anti-rat Alexa Fluor 594 (1:1000, A21209, Invitrogen) or donkey anti-mouse Alexa Fluor 488 (1:500, A-21202, Invitrogen). Cells were washed and then incubated in DAPI (1:1000, from a stock of 1ul/ml, Sigma-Aldrich, UK) diluted in 2 % DS in 0.02 % PBS-T followed by washing with PBS. Coverslips were mounted onto slides using DAKO Fluorescence mounting medium and slides kept at 4°C until imaging.

**Experimental design of *in vivo* experiments**

***Effects of acute SAFit2 administration in the forced swim test and on plasma corticosterone***

*Forced swim test*

The effects of acute SAFit2 administration on antidepressant-like behavior was evaluated using the forced swim test (FST) (5-7). 8-week old C57Bl6 male mice were injected (i.p.) with either SAFit2 (20 mg/kg) or vehicle [4 % (v/v) ethanol (Sigma-Aldrich, UK) , 5 % (v/v) PEG-400 (Fisher Scientific, Ireland) and 5 % (v/v) Tween 80 (Sigma-Aldrich, UK) in saline] 16 h or 1 h prior to the FST. Briefly, mice were individually placed in a clear glass cylinder (24 × 21 cm) filled with water (23–25 °C) to a depth of 17 cm and allowed to swim for 6 min. At the end of the trial, mice were dried and placed back into their home cage. The test was digitally recorded, and the time spent immobile during the last 4 min of the 6 min trial was measured. Immobility was defined as the total absence of movement except slight motions to maintain the head above the water.

*Plasma corticosterone measurement*

At baseline, immediately prior to the start of the FST, tail blood was collected. A single edge razor blade was used to make a 2–4 mm long diagonal incision in the tip of the tail and EDTA-containing capillaries (BD diagnostics) were used to collect blood. 30 and 90 min after the FST had started, blood was sampled again using the same incision. Approximately 40 μl of whole blood was taken per time point, centrifuged at 2500 g for 15 min at 4°C. Plasma was removed and kept at -80°C until analysis.

Plasma corticosterone was measured using a commercial ELISA kit (ADI-901-097, Enzo) according to the manufacturer´s protocol. Light absorbance was read with a multi-mode plate reader (Synergy HT, BioTek Instruments, Inc.) at 405 nm. Samples were analyzed in duplicate within a single assay and the minimum threshold of detection was less than 32 pg/mL. The limit for the coefficient of variation used was 20 % and the final concentrations are expressed in ng/mL.

**Effects of chronic SAFit2 or fluoxetine administration on stress-induced changes in behavior.**

The experimental design is summarized in figure 5A.

*Drug administration*

8-week-old C57Bl6 male mice were single housed 1 week before starting the stress paradigm. One day prior to initiation of stress paradigm and drug administration, mice received four BrdU injections (7.5 mg/mL in Saline; 75 mg/kg, i.p., Sigma Cat# B5002) at 2-hour intervals (6, 8). Animals were randomly assigned to each experimental condition.

Stressed and non-stressed animals were administered SAFit2 (20 mg/kg; i.p.) or Vehicle (i.p.) twice a day or Fluoxetine (10 mg/kg, i.p.; Sigma-Aldrich, UK) once a day. Fluoxetine was included to compare SAFit2 phenotypic output to that of a known antidepressant. SAFit2 was dissolved in Vehicle [4 % EtOH (Sigma-Aldrich, UK), 5 % Tween 80 (Sigma-Aldrich, UK) and 5 % PEG 400 (Fisher Scientific, Ireland) in 0.9 % saline] and fluoxetine in Saline. Drugs were prepared fresh every two days. Body weight recording and dosing adjustment were done daily. Morning injection took place before 9 am and at least 2 h before the start of the stressor or any behavioral testing. The second injection was administered after 6 pm. There were five experimental groups: non-stressed animals injected with vehicle (n=9); non-stressed animals injected with SAFit2 (n=9); stressed animals treated with vehicle (n=10) or stressed animals treated with SAFit2 (n=10) and stressed animals treated with Fluoxetine (n=10).

*Chronic Psychosocial Stress*

Animals underwent 5 weeks of psychosocial stress with social defeat and overcrowding (7, 9, 10). For the social defeat procedure, age matched male CD1 mice (n = 35; Harlan, UK) were tested for aggression on three individual days. Briefly, CD1 mice were exposed to another CD1 intruder mouse in their home cage until the first attack commenced. The 30 CD1 mice with the shortest attack latencies were used for the social defeat procedure, while the rest were used in the social interaction test. Test mice undergoing psychosocial stress were assigned to a different CD1 mouse on each day.

Over a 35-day period, C57BL/6J mice were stressed according to a temporally unpredictable mixed schedule of social defeat and overcrowding sessions. During the social defeat procedure, stress mice were gently placed in the home cage of the CD1 aggressor and allowed to interact until the first attack of the CD1 mouse took place, which was followed by a defeat posture of the stress mouse. Mice were then separated for 2 h by a perforated Plexiglass divider allowing for auditory, olfactory and visual, but not physical contact. The divider was subsequently removed, after which another social defeat took place, and test mice were placed in their original home cage. For the overcrowding procedure, stressed mice of one group (n = 8 - 10) were housed in a standard holding cage for 24 h. During the 5 weeks, non-stress animals were handled daily. No stress took place the days that animals underwent behavioral testing.

*Behavioral tests*

Behavioral assessment took place during the last two weeks of the experiment (Fig 5A). Behavioral tests were performed in the following order: (1) social interaction test; (2) open field; (3) novelty induced hypophagia, (4) female urine sniffing; (5) light-dark box and (6) forced swim test.

*Social interaction test*

The social interaction test was used to assess avoidance of the CD1 aggressors and was conducted as previously described (10, 11). The test was performed in an open arena (40 × 32 × 24 cm, L × W × H) containing an empty wire mesh cage (9.5 × 7.5 × 7.0 cm) within an area termed the interaction zone. The test consisted of two 2.5 min trials, with a 1 min intertrial interval. In the first trial, the wire mesh cage was left empty, whereas an unfamiliar CD1 aggressor was placed in the wire mesh cage in the second trial. C57BL/6 mice were allowed to explore the arena freely during trials and both mice were returned to their homecage after the test. In between animals, the arena was cleaned with 70 % ethanol. To reduce potential anxiogenic factors, all mice were habituated to the testing room for 1 h before testing, and testing was conducted under red light (5 lux). All trials were videotaped using a ceiling camera and analyzed for time spent in the interaction zone using Ethovision version 13 software (Noldus). Social avoidance was calculated by dividing the time spent in the interaction zone in the presence of the CD1 mouse (trial 2) by the time spent in the interaction zone when the wire mesh was empty (trial 1).

*Open field test*

Mice were assessed for their locomotor activity and response to a novel environment in the open field test, which was conducted as previously described (10). Animals were habituated to the testing room 1 h before the test. Mice were placed in an open arena (45 × 45 × 45 cm, L × W × H) and were allowed to explore the arena for 10 min. Testing was performed under dim light (60 lux). Experiments were videotaped using a ceiling camera and were analyzed for time spent in the center zone (defined as 50 % away from the edges) and total distance travelled. Data was analyzed using Ethovision version 13 software (Noldus) in two 5 min blocks, to separate an early exploration phase from behavior after habituation.

*Novelty induced Hypophagia*

The novelty-induced hypophagia test is a test of anxiety sensitive to chronic antidepressant treatment (12, 13). Briefly, mice were trained to drink a diluted solution of sweetened condensed milk (3:1, water to milk) from a 10 ml serological pipette through the lid of their cage for 30 min per day for 3 days. On the fourth day, the latency to drink the milk in their homecage was measured. On the fifth day, mice were placed in a novel brightly lit cage (1200 lux) without bedding, and their latency to drink the milk was recorded. The data are presented as latency difference (i.e. the latency to drink in the novel cage minus the latency to drink in the home cage).

*Female urine sniffing test*

Mice were assessed for hedonic and reward-seeking behavior in the female urine sniffing test, which was performed as previously described (9). Prior to starting the sniffing test, vaginal smears from age-matched female C57Bl/6 mice (n = 15; Envigo, UK) were taken to assess the estrous cycle. Urine from female mice in the estrous stage was collected and pooled. Male mice were brought to the testing room and habituated to the test by presenting a cotton bud attached to the lid of their housing cage for 45 min before the start of the test. Test mice were subsequently introduced to a new cotton bud containing 30 μl sterile water for 3 min. After a 45 min intertrial interval a new cotton bud containing 30 μl urine from a female mouse in estrous was presented to the mouse for 3 min. All stages of the experiment were conducted in red light (5 lux). All tests were videotaped using a ceiling camera for 3 min and interaction time with the cotton bulbs was scored blinded.

*Light-dark box*

Mice underwent the light-dark box to evaluate anxiety-like behavior (14, 15). The apparatus consisted of a clear plexiglass cage (44 x 21 x 21 cm, L x W x H) separated into two compartments by a partition, which had a small opening (10 x 5 cm) at the floor level. The open compartment was made of transparent plexiglass and brightly illuminated with white light of 1000 lux intensity. The smaller compartment was 14 m long and made from black plexiglass. Mice were individually placed in the center of the brightly lit compartment, facing away from the partition and allowed to freely explore the apparatus for 10 minutes. At the end of the trial, mice were placed back into their home cage and the apparatus was cleaned thoroughly. The number of light-dark transitions were recorded by a blind observer (16, 17).

*Forced swim test*

The forced swim test was used to assess antidepressant drug-like activity (18, 19). It was performed and scored as described above.

*Plasma corticosterone measurement*

Blood was taken at two timepoints during the chronic stress paradigm to evaluate the effect of stress and drug treatment on plasma corticosterone concentrations. On day 17 of the experiment, blood was collected by a nick in the medial part of the tail. On day 35, the last day of experiment, blood was collected from end of tail 5 minutes before and 30, 60 and 120 min after the FST. Preparation of plasma and the measurement of corticosterone measurement was conducted as described above.

*Transcardial perfusion and brain sectioning*

On day 35 and 3 h after FST, animals were deeply anaesthetized with sodium pentobarbital (90 mg/kg), and transcardially perfused with PBS followed by 4% Paraformaldehyde (PFA). The brains were post-fixed in cold 4% PFA for 24 hours at 4°C and cryoprotected in sucrose gradient (15% sucrose solution for 24 h and 30 % sucrose solution for 24 h at 4°C) before being snap-frozen and stored at -80°C. The brains were then sectioned at 35 µm using a Leica CM1900 cryostat and stored in cryoprotectant at -20°C until further processing.

*Immunohistochemistry of hippocampal sections*

Dorsal (-1.55 mm to -2.03 mm to the Bregma) and ventral (-2.79 mm to -3.27 mm to the Bregma) hippocampal sections were stained for DCX+ neuronal cell bodies to m immature neuron formation in this area. Sections were placed in 10 mM PBS for 5 min to rehydrate, then blocked with 10 % DS in 0.3 % PBS-T for 2 h at room temperature. Sections were then incubated in primary rabbit anti-DCX (1:500, ab18723, Abcam) in 2 % DS, 0.3 % PBS-T, overnight at room temperature. The next day, sections were washed in PBS with 0.3 % PBS-T and incubated in donkey anti-rabbit secondary antibody (1:200, Alexa Fluor 488) in 2% DS, 0.1% PBS-T for 90 min at room temperature and protected from light. Sections were then washed in PBS and counterstained with DAPI for 5 min at room temperature, in the dark. Lastly, sections were washed in PBS, mounted on glass slides and cover-slipped with PVA DABCO mounting media. Slides were stored at 4°C and covered from the dark until imaging.

**Fluorescence microscopy and image analysis**

Cell and hippocampal slides were viewed under an Olympus BX53 upright microscope and photomicrographs of immunopositive cells were captured at 20x or 40x magnification using an Olympus DP71 camera and CellSens™ capture software. Exposure time was kept the same across treatments. Investigators who performed quantification were blinded to treatment groups.

Immunocytochemistry: For each treatment condition, 5 fields of view were randomly captured per coverslip, there were 3 coverslips per treatment per plate, and each experiment was repeated 3 times. NeuronJ plugin of ImageJ 1.44 (20) was used to measure the length of dendritic processes. Traced neurites were labeled as primary (emanating directly from the soma), secondary (branching from a primary), or tertiary (branching from a secondary). Total length of neurites (all neurites) and number of bifurcations (nodes) were measured. The number of neurons (DCX+ or β3-tubulin+ ) with a primary [(neurons with primary processes/total number of neurons) x 100], a secondary process [(neurons with secondary processes/total number of neurons) x 100] and a tertiary process [(neurons with tertiary processes/total number of neurons) x 100] were also calculated. The CellCounter plugin of ImageJ 1.44 was used for counting DAPI, BrdU and β3-tubulin+ cells. The percentage of differentiated neurons [(β3-tubulin cells/total DAPI+ nuclei) x 100] was calculated. In the proliferation experiments, the percentage of proliferating cells [(BrdU+ cells/DAPI cells) x 100] were calculated.

Immunohistochemistry: For each treatment group, 3 hippocampal sections were randomly captured per dorsal hippocampus and per ventral hippocampus, and 5-6 animals were used per experimental group. DCX+ cells were counted manually. For whole hippocampus measurement, dorsal and ventral quantification per animal were added.

**Statistical analysis**

Data is shown as mean + SEM. Statistical analysis was performed using IBM SPSS Statistics 26. *In vitro* data and the latency to social defeat from *in vivo* were analyzed using one-way ANOVA, followed by Tukey *post hoc* test for group-wise comparisons when appropriate. Student’s *t-*test was used to analyze the *in vivo* acute SAFit2 administration experiment. Kruskal-Wallis non-parametric test followed by Dunn’s comparison was used to analyze the behavioral data of the chronic stress and SAFit2 administration in the *in vivo* experiment. DCX+ cell bodies per section of the dentate gyrus were analyzed using two-way ANOVA followed by Tukey post hoc test for group-wise comparisons when appropriate. For all comparisons, p<0.05 was the criterion used for statistical significance.

**Supplementary results
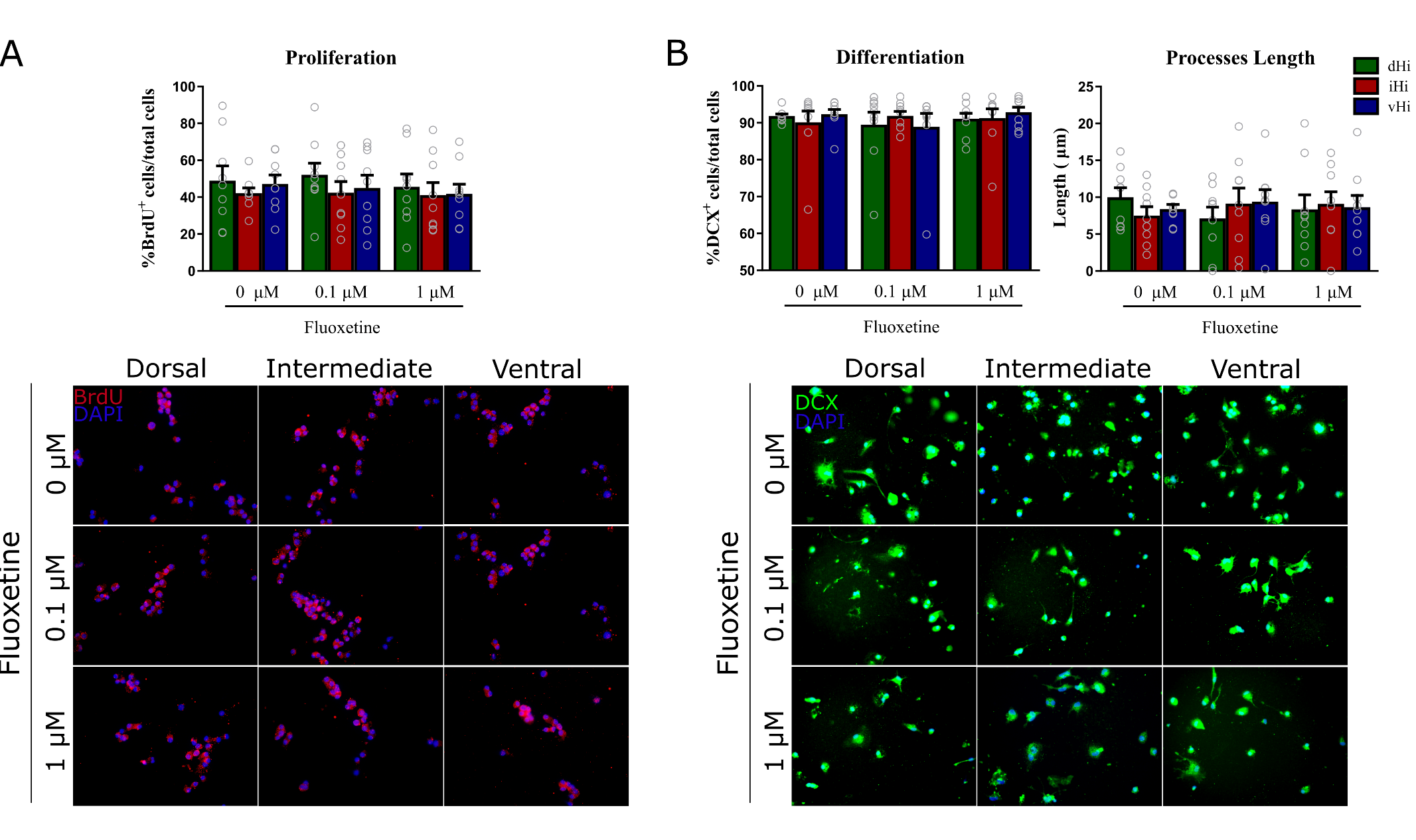
**

**Supplemental Figure 1:** Effects of fluoxetine exposure on differentiation and proliferation of NPC derived from dorsal (dHi), intermediate (iHi) and ventral (vHi) hippocampus. For proliferation (A), NPCs were isolated and cultured as neurospheres for 4 DIV, then dissociated and treated with fluoxetine (0.1 µM or 1 µM) for 4h *in vitro* prior to fixation and immunocytochemistry. Fluoxetine did not affect cell proliferation (BrdU+ cells) in NPCs derived from the dHi, iHi or vHi. Representative images of proliferating cells derived from the dHi, iHi and vHi are shown; stained with DAPI (blue) and BrdU (red). For differentiation (B), neurospheres were dissociated after proliferating for 4 days and exposed to fluoxetine for further 4 DIV under differentiation conditions prior to fixation and immunocytochemistry. 4 DIV exposure to fluoxetine (0.1 µM or 1 µM) had no effects on neuronal differentiation of NPCs derived from either the dHi, iHi or vHi . Representative images of immature neurons differentiated from dHi, iHi and vHi NPCs are shown; stained with DAPI (blue) and DCX (green).

**
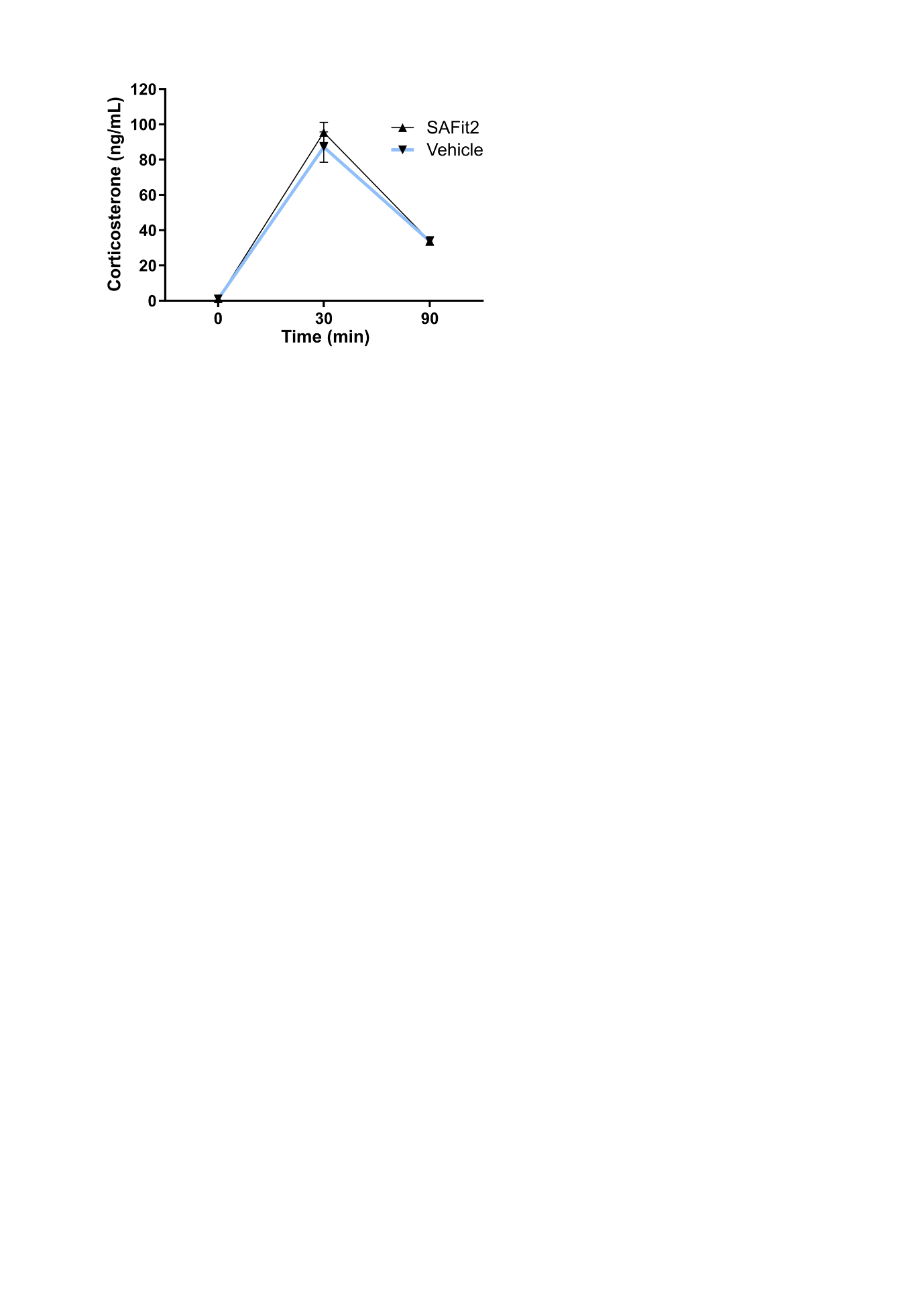
**

**Supplemental Figure 2:** Effect of acute SAFIT2 administration on plasma corticosterone concentrations. 16 hours after i.p. administration of SAFit2, plasma was sampled before and after FST (forced swim test). While the inhibitor decreased immobility time in FST (Fig 4 in manuscript), it did not alter plasma corticosterone response to acute stress. mean + SEM, n= 7-8 per group.


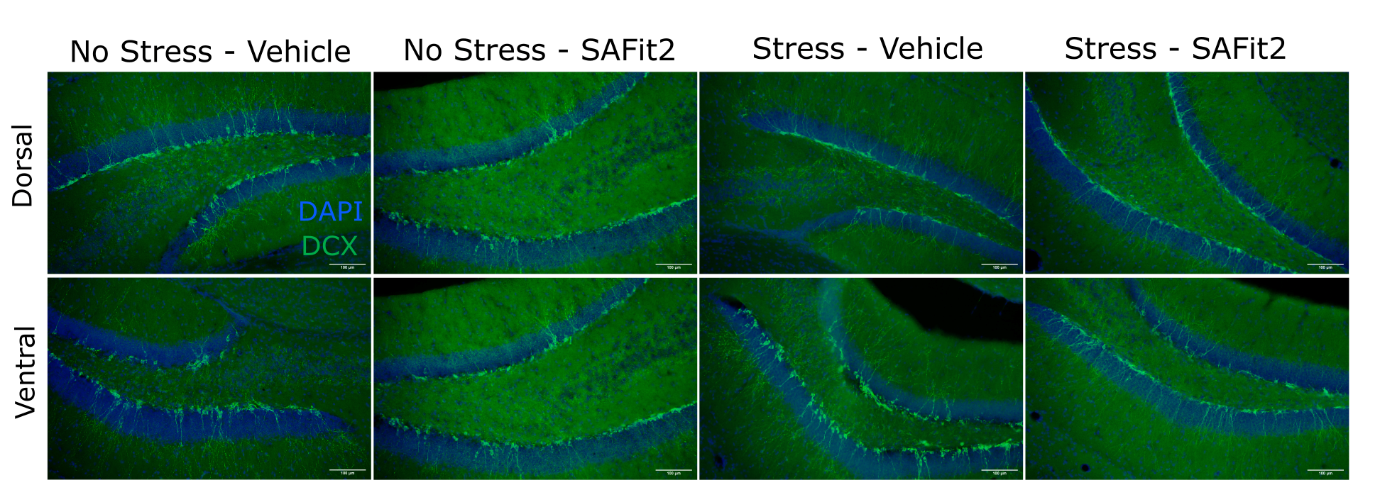


Supplemental Figure 3. Representative images of dentate gyrus DCX+ straining in stressed and SAFIT2 treated animals. New immature neurons are green and counterstained with DAPI (blue). Scale: 100 µm

**
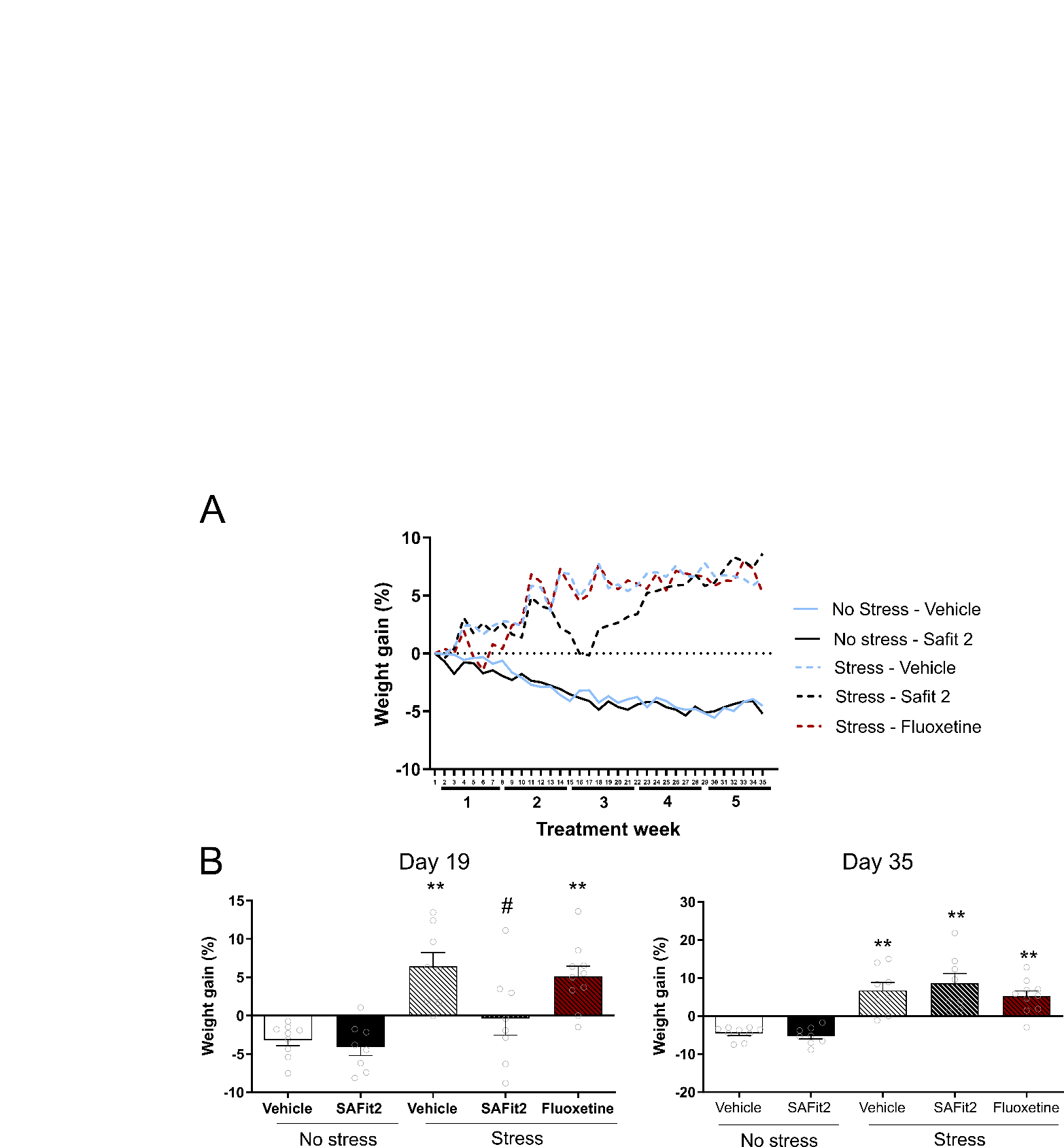
**

**Supplemental Figure 4.** Effect of chronic SAFit2 on stress-induced weight gain trajectory. While Vehicle and Fluoxetine -treated stressed animals showed a steady increase in weight gain, those treated with SAFit2 showed a distinctive weight gain trajectory (A). After 19 days of stress, animals treated with the inhibitor showed no weight gain while stress-induced weight gain was already evident in Vehicle and Fluoxetine treated animals (C). By the end of the experiment all stressed groups showed a significant increase in weight gain . mean + SEM, n= 8-10, ** p<0.01 vs no stress-vehicle and #p<0.05 vs stress-vehicle by Kruskal-Wallis followed by Dunn’s comparison.

**References**

1. Gaali S, Kirschner A, Cuboni S, Hartmann J, Kozany C, Balsevich G, et al. Selective inhibitors of the FK506-binding protein 51 by induced fit. Nat Chem Biol. 2015;11(1):33-7.

2. Hartmann J, Wagner KV, Gaali S, Kirschner A, Kozany C, Rühter G, et al. Pharmacological Inhibition of the Psychiatric Risk Factor FKBP51 Has Anxiolytic Properties. The Journal of neuroscience : the official journal of the Society for Neuroscience. 2015;35(24):9007-16.

3. Quintá HR, Maschi D, Gomez-Sanchez C, Piwien-Pilipuk G, Galigniana MD. Subcellular rearrangement of hsp90-binding immunophilins accompanies neuronal differentiation and neurite outgrowth. J Neurochem. 2010;115(3):716-34.

4. Levone BR, Codagnone MG, Moloney GM, Nolan YM, Cryan JF, O’ Leary OF. Adult-born neurons from the dorsal, intermediate, and ventral regions of the longitudinal axis of the hippocampus exhibit differential sensitivity to glucocorticoids. Molecular Psychiatry. 2020:1 - 13.

5. Cryan JF, Dalvi A, Jin S-H, Hirsch BR, Lucki I, Thomas SA. Use of Dopamine-β-hydroxylase-Deficient Mice to Determine the Role of Norepinephrine in the Mechanism of Action of Antidepressant Drugs. Journal of Pharmacology and Experimental Therapeutics. 2001;298(2):651.

6. Felice D, O'Leary OF, Pizzo RC, Cryan JF. Blockade of the GABAB receptor increases neurogenesis in the ventral but not dorsal adult hippocampus: relevance to antidepressant action. Neuropharmacology. 2012;63(8):1380-8.

7. van de Wouw M, Boehme M, Lyte JM, Wiley N, Strain C, O'Sullivan O, et al. Short-chain fatty acids: microbial metabolites that alleviate stress-induced brain–gut axis alterations. The Journal of Physiology. 2018;596(20):4923-44.

8. O’Leary OF, O’Connor RM, Cryan JF. Lithium-induced effects on adult hippocampal neurogenesis are topographically segregated along the dorso-ventral axis of stressed mice. Neuropharmacology. 2012;62(1):247-55.

9. Finger BC, Dinan TG, Cryan JF. High-fat diet selectively protects against the effects of chronic social stress in the mouse. Neuroscience. 2011;192:351-60.

10. Burokas A, Arboleya S, Moloney RD, Peterson VL, Murphy K, Clarke G, et al. Targeting the microbiota-gut-brain axis: prebiotics have anxiolytic and antidepressant-like effects and reverse the impact of chronic stress in mice. Biological psychiatry. 2017;82(7):472-87.

11. O’Leary OF, Felice D, Galimberti S, Savignac HM, Bravo JA, Crowley T, et al. GABA<sub>B(1)</sub> receptor subunit isoforms differentially regulate stress resilience. Proceedings of the National Academy of Sciences. 2014;111(42):15232.

12. O’Leary O, Zandy S, Dinan T, Cryan J. Lithium augmentation of the effects of desipramine in a mouse model of treatment-resistant depression: a role for hippocampal cell proliferation. Neuroscience. 2013;228:36-46.

13. Dulawa SC, Hen R. Recent advances in animal models of chronic antidepressant effects: the novelty-induced hypophagia test. Neuroscience & Biobehavioral Reviews. 2005;29(4-5):771-83.

14. Jacobson LH, Bettler B, Kaupmann K, Cryan JF. Behavioral evaluation of mice deficient in GABA B (1) receptor isoforms in tests of unconditioned anxiety. Psychopharmacology. 2007;190(4):541-53.

15. O’Mahony CM, Sweeney FF, Daly E, Dinan TG, Cryan JF. Restraint stress-induced brain activation patterns in two strains of mice differing in their anxiety behaviour. Behavioural brain research. 2010;213(2):148-54.

16. Crawley JN. Neuropharmacologic specificity of a simple animal model for the behavioral actions of benzodiazepines. Pharmacology Biochemistry and Behavior. 1981;15(5):695-9.

17. Holmes A, Iles J, Mayell S, Rodgers R. Prior test experience compromises the anxiolytic efficacy of chlordiazepoxide in the mouse light/dark exploration test. Behavioural brain research. 2001;122(2):159-67.

18. Porsolt RD, Le Pichon M, Jalfre M. Depression: a new animal model sensitive to antidepressant treatments. Nature. 1977;266(5604):730-2.

19. Cryan JF, Mombereau C. In search of a depressed mouse: utility of models for studying depression-related behavior in genetically modified mice. Molecular psychiatry. 2004;9(4):326-57.

20. Meijering E, Jacob M, Sarria JC, Steiner P, Hirling H, Unser M. Design and validation of a tool for neurite tracing and analysis in fluorescence microscopy images. Cytometry Part A: the journal of the International Society for Analytical Cytology. 2004;58(2):167-76.
